# Everybody's Page

**Published:** 1888-10-20

**Authors:** 


### Everybody's Page.

"The more vigorous are a man's physical movements, the less need has he of ablutions for the maintenance of his health, partly because the continued friction of most parts of the skin and the perspiration produced by work are sufficient in themselves to perform the mechanical task of cleansing. Thus experience teaches that to the labourer baths and ablutions are less necessary than to a man of a sedentary mode of life, and to the adult than to the infant, whose lungs discharge their office feebly."

Stammering depends neither on structural defects of the mouth, nor on imperfect education or bad habits, nor on paralysis of the organs concerned in speech. Nor, again, is it the result of mental defect. It is a peculiar affection of speech, in which, in the course of utterance, a sudden spasm occurs, which either checks the flow of sound altogether for a short space, or in which the arrest of speech is attended with a series of rapidly-repeated sounds, which are usually those literal sounds at which the check took place.

"Fast" dyes—*i.e.*, those which do not fade—are more wholesome than dyes which fade readily. The fading is generally, although not always, caused by evaporation. A dye which fades rapidly, therefore, gives off a concentrated vapour, and is thus injurious. A dye which fades or evaporates little, gives off an attenuated vapour, and may, therefore, be more wholesome. Whether a colour will fade quickly or slowly will partly depend upon the volatility of the colouring matter, as also on whether the latter simply rests upon the fibre or saturates it. The foregoing explains the important difference between indigo black and other black dyes; the former, as a much "faster" dye, is considerably less injurious than the rapidly-fading logwood dyes. Among the lighter colouring matters, cochineal is notoriously the "fastest," and accordingly it is most wholesome.

At one time, in the Asylum of the House of Industry, in Dublin, scrofula prevailed in so great a degree that it was generally believed that the disease was contagious. This it was not, however; but the wards were so crowded with children that the air became impure to a degree. In one ward of moderate height, 60 feet long by 18 broad, were 38 beds, most of which contained three children, the entire amounting to upwards of 100. The matron of this asylum remarked that "there was no enduring the air of this apartment when the doors were first thrown open in the morning," and that "it was in vain to raise any of the windows, as those children who happened to be inconvenienced by the cold closed them as soon as they had an opportunity." As the air they breathed in the daytime was but little better, there is no occasion for wonder at the general spread of the disease amongst them.

One of the most interesting defects of speech to the physician is that to which the name of aphasia is generally applied. It presents many varieties, both in kind and in degree. In one form, which is comparatively rare, the patient loses absolutely the power of uttering any articulate sound, though probably apparently well in every other respect. He hears and understands all that is said to him; he sees and comprehends all that he reads; he can communicate his ideas by writing as well as ever he did; he can open and shut his mouth, can move his tongue freely, can masticate and swallow without impediment; he can, in fact, use his muscles of articulation for every other purpose than articulation with perfect ease and facility—yet, if he be asked to speak, if he be asked to read, he is as much at a loss to utter even a single articulate sound as a man who had never seen or heard of a telegraph apparatus would be to send messages by means of it.

An African clergyman, in preaching from the text: "And multitudes came unto Him, and He healed them of divers diseases," said: "This is a terrible text, my dying congregation. Disease is in the world. The small-pox slays its hundreds, the cholera its thousands, and yellow fever its tens of thousands, but in the language of our text, if you take the divers, you are gone. These earthly doctors can cure small-pox, cholera, and yellow fever, if they get there in time, but nobody but the good Lord can cure the divers."

Mr. A. J. Buhay reports, in the *Edinburgh Medical Journal*, the account of a favourable experience with a 20 per cent. solution of menthol in laryngeal and pulmonary phthisis. The solution is injected with a special syringe into the larynx or trachea with the aid of a laryngeal mirror. In the case of laryngeal phthisis, two or three injections, of 15 minims each, are made at each sitting. Deep inspiration should follow the injection, whereby the anæsthetic effect is increased. No untoward effects follow, and the treatment should be repeated once or twice daily for about two months. If possible the patient should hourly, during the day, inhale five or more minims of the same solution added to a pint of boiling water or to a pledget of cotton-wool placed in a respirator. The treatment, it is said, causes pain and discomfort in the larynx to disappear, and relieves dysphagia. Ulceration ceases and smooth cicatrices result.

Attention was first directed to the remarkable development of myopia during school life by Jäger, in 1861, whose observations were, however, made upon comparatively few children. The subject was soon afterwards taken up by Cohn, of Breslau, who examined more than 10,000 scholars, and who was able to deduce some very remarkable conclusions from his observations; for he found, in the first place, that the children attending rural schools were almost free from short-sightedness, but that the affection increased in frequency in proportion to the demands made upon them, attaining its maximum in the gymnasia; secondly, that the number of myopic children, proceeding from the lower to the higher classes in all schools, was augmented in a regular and uniform manner; and lastly, that the degree of myopia increased from class to class, so that not only were there more myopic youths of both sexes in the higher classes, but the imperfection of vision consequent on the degree of myopia was much greater in the higher classes than in the lower.

The only quite safe bath for an ordinary man or woman, without training, for all the purposes of cleanliness, and for bringing on the reaction, is cold sponging, either with or without previous hot sponging. The process can be varied in many ways, according to the feelings and state of health of the bather, and no one need cease to have baths altogether because too delicate for the common practice, or because there is not a bath-room in the house. All that is required is an ordinary portable bath or a large "mug." A small quantity of warm water may be put into the bath, just enough to keep the feet warm. Then the cold or cool water supplied from a basin or the tap is to be applied by a sponge over the head and neck, limbs and trunk, as quickly as possible. The exertion required for this is an advantage. A final application of the sponge to the nape of the neck, in order to send a stream down the hollow of the spine, may complete the process. Everything, including the process of drying, should be done without loss of time. This is all that is necessary for health. A man who is ambitious and very robust may reach the profoundest depth of a plunge bath without a fit of apoplexy resulting in his youth, but there is no evidence to show that he is better off than his less ambitious contemporary who acts more cautiously.

